# Carbon-based sprayed electrodes for pyroelectric applications

**DOI:** 10.1371/journal.pone.0221108

**Published:** 2019-08-15

**Authors:** C. Chirila, M. Botea, A. Iuga, A. G. Tomulescu, L. Balescu, A. C. Galca, A. G. Boni, L. Leonat, I. Pintilie, L. Pintilie

**Affiliations:** National Institute of Materials Physics, Magurele-Ilfov Romania; Universiti Malaysia Pahang, MALAYSIA

## Abstract

A carbon-based layer was deposited by spraying on top of a ferroelectric layer grown by sol-gel on Si (001) substrate and its properties as electrode and absorber for pyroelectric detection were tested. It was found that the electric properties of the ferroelectric capacitor with top carbon-based sprayed electrode (CBSE) are comparable with those of the capacitors with standard top SrRuO_3_ (SRO)/Au electrode. Pyroelectric measurements show that the pyroelectric signal recorded on ferroelectric capacitors with top CBSE electrode is 2.5 times greater than for top SRO/Au electrode for low frequency range. The value of the pyroelectric coefficient was estimated to 9.73·10^−4^ C/m^2^K for CBSE electrodes and 3.36·10^−4^ C/m^2^K for SRO/Au respectively. The fabrication process of CBSE is of low cost, easy to implement and with high throughput making it attractive for manufacturing various devices like pyroelectric detector, thermal imaging, solar cells, etc.

## Introduction

“Internet of things” electronic devices follow closely the miniaturization, functionality and portability trends, and require for their future development a self-sufficient power supply in the form of energy storage or harvesting devices. For this reason pyroelectric materials are important candidates in the energy conversion field due to their capability to generate power by using temporal temperature changes as predicted by early studies [1,2]. Recently, notable progress has been achieved to enhance the performance of pyroelectric materials and their integration on different devices, for instance sensors, detectors, imaging tools, etc. Coupling the thermal and the solar energy harvesting is another promising area were pyroelectric materials demonstrate their potential for new applications [3].

From the multitude of pyroelectric materials, lead zirconate-titanate (PZT) is the most used material for applications based on the pyroelectric effect, such as infrared (IR) detectors and, more recently, for energy harvesting. This is due to the high value of the pyroelectric coefficient, leading to enhanced energy conversion efficiency [4–6]. The properties of the top electrode, exposed to IR radiation, are also important for pyroelectric applications. This should have excellent electrical and thermal conductivity, allowing fast transfer of charges and heat, as well as good absorption of the IR radiation, allowing a larger temperature variation of the pyroelectric element [7,8]. Carbon coatings as well as carbon-based composites are good candidates both for absorber layer and for electrodes in pyroelectric applications. For example, graphene and graphene based composites were produced by various methods, such as screen printing, chemical-vapour deposition, self-assembling or wet spinning to improve the electrode properties and integrated in pyroelectric harvesters [9–12]. Also, carbon nanotubes were considered for absorber layers to cover the top metal electrode of pyroelectric elements used for manufacturing IR detectors. [8,13,14]. However, both graphene and carbon nanotubes are relatively expensive materials and the methods used to deposit carbon-based absorbers or electrodes are in many cases time consuming and relatively expensive (e.g. vapour deposition, screen printing, electro-spraying) [9–14]. Simpler techniques can be also used to deposit carbon layers, for example spraying combined with spin coating, or a candle soot coating, leading to significant improvement of the IR sensitivity of the pyroelectric elements whether in the form of thin films or bulk [15,16].

In this paper we investigate the performances of carbon-based sprayed electrodes (CBSE), as top contact on the surface a PZT/SRO/STO/Si structure, for pyroelectric applications and with the PZT film deposited by sol-gel method. The aim is to establish a low cost manufacturing method of pyroelectric elements, without degrading the characteristics of PZT-based pyroelectric detectors. Simple spray deposition is a very appealing fabrication method due to its advantages, like the possibility to deposit a carbon layer on large surfaces, use of commercial carbon sources, like carbon paste, use of nontoxic solvents for the carbon paste, possibility to control the thickness, and compatibility with industrial manufacturing.

## Experimental

### Deposition of the PZT layer

Lead zirconate-titanate (PZT) layers with Zr/Ti ratio of 20/80 were deposited by sol-gel technique on Si (001) substrates buffered with SrTiO_3_ (STO) and SrRuO_3_ (SRO) layers. The STO layer plays the role of a template for textured or epitaxial growth of the PZT, while SRO film plays the role of the bottom electrode for the ferroelectric capacitor. The methods and the parameters used for the deposition of the STO and SRO layers are presented elsewhere [17–19]. The solution for the deposition of the PZT film with pyroelectric properties has a concentration of 0.5 M and was obtained from the following precursors: lead acetate trihydrate Pb(CH_3_COO)_2_ 3H_2_O (Sigma-Aldrich, 99%), zirconium n-propoxide Zr[O(CH_2_)_3_CH_3_]_4_ (Sigma Aldrich, 70 wt% solution in 1-propanol), and titanium isopropoxide Ti[OCH(CH_3_)_2_]_4_ (Sigma Aldrich, 99%). The precursors were dissolved in 2-methoxyethanol in inert atmosphere (N_2_). The obtained sol was deposited by spin-coating on the Si/STO/SRO support (3000 rpm for 20 s), followed by a first pyrolysis step to remove the solvent (at 200°C for 2 min) and a second pyrolysis step to burn the organic residues (at 400°C for 4 min). The crystallization of the film was carried out in air by conventional thermal treatment in a tubular furnace, at 650°C for 60 min, with a heating rate of 10°C/min.

The PZT films were further used to manufacture the ferroelectric capacitors by depositing a top electrode. The top electrode was either SRO/Au or a carbon layer. The deposition of the top SRO/Au contacts through a shadow mask is described elsewhere [17]. The Au layer was deposited just to make visible the SRO contacts. The deposition of the carbon-based electrodes is described in the next section. The area of the top contacts was of 0.2 mm^2^ in both cases.

### Deposition of carbon-based sprayed electrodes

**Fig 1:**
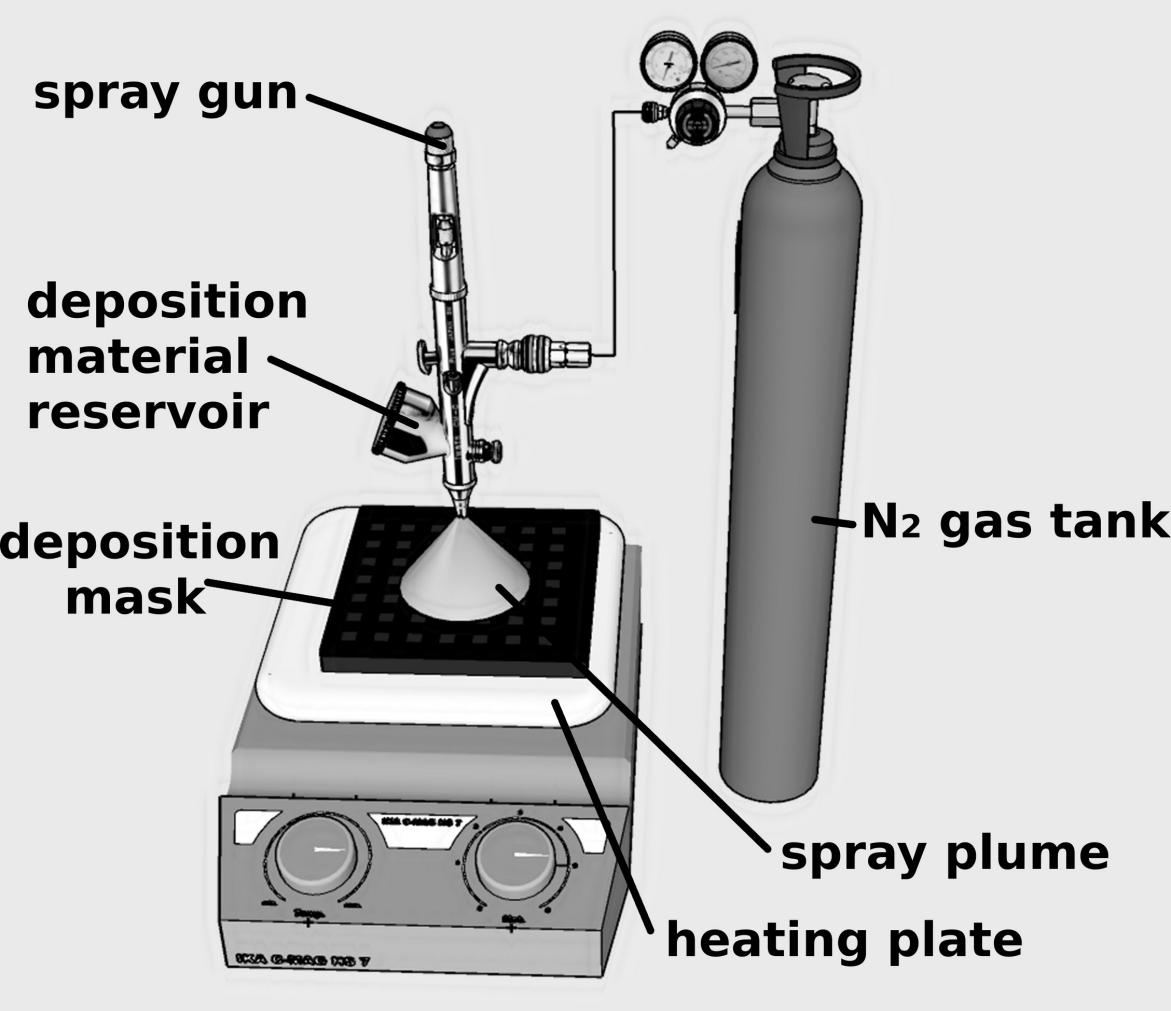
Schematic representation of CBSE deposition process.

Spray deposition consists in the pulverization of a material (in the form of a solution or a dispersion), using a carrier gas, through a spray nozzle onto a preheated substrate to evaporate the solvent. Multiple parameters like the type of solvent, solution concentration, carrier gas type and pressure, substrate temperature, nozzle-sample distance and/or diameter, etc. can be varied in order to control the thickness and properties of the film. The schematic diagram of the experimental setup used to fabricate carbon-based electrodes by spray deposition is depicted in (Fig 1) and it contains a spray gun with 0.2 mm nozzle and a deposition material reservoir, a connection to a N_2_ gas tank that acts as a carrier gas supply and a heating plate. The sample is placed onto the heating plate with a deposition mask, having the desired geometry, mounted on top.

Commercial carbon paste (Electrodag PF-407C from Loctite) was used for the fabrication of CBSE. To obtain the proper carbon dispersion, different solvents were tested (eg.: water, ethylic alcohol, acetone, trichloroethylene) and it was found that acetone was the most appropriate. As such, dispersion with 1:10 (volume ratio) carbon paste to acetone was prepared by sonication in an ultrasonic bath for 15 minutes. Nitrogen was chosen as a carrier gas.

Parameters such as N_2_ pressure, the distance between the nozzle and the mask surface, spray time and substrates temperature were varied to obtain well defined electrodes with good mechanical and conductive properties. The optimal nozzle-to-mask distance was found to be ~ 15 cm. Shorter distances lead to irregular electrodes; although the drying time in this case was longer, and/or the sample temperature was higher than 120 ^o^C, when the mask was removed, part of the carbon electrodes remained glued to the mask, leading to non-uniform electrodes with poor mechanical properties. When the distance between the nozzle and mask was increased the droplets dried completely before colliding with the substrate, leading to a poor adhesion and failed deposition. The same effect was observed when the N_2_ pressure was higher than 1.5 bar. In order to obtain uniform CBSE electrodes with good adhesion and conductive properties the sample was heated at 120 ^o^C, the distance between nozzle-to-mask was maintained at 15 cm and the N_2_ pressure was set at 1.5 bar; after deposition, the samples were maintained on the hot plate at the deposition temperature for 15 minutes.

### Experimental methods

X-ray diffraction studies were performed using an D8 ADVANCE (BRUKER-AXS) equipment dedicated to thin film analysis for structural phase determination. Detailed structural information was obtained by performing symmetric scans in relation to Si (001) plane (2Theta-Omega scan), around reflections of different orders of these planes. The alignment of the substrate by the cut-off correction results in an increase in the height of the substrate lines associated with the decrease of the width, allowing for a more accurate determination of the position and the real profile of the diffraction peaks.

The specular reflectance measurements have been done by employing a Woollam V-VASE spectroscopic ellipsometer equipped with a HS-190 monochromator. The angles of incidence and reflection are chosen to the minimum allowed by the equipment, n.a. 25°. The measurements were done with both p- polarized and s- polarized incoming light beam, resulting in pR and sR dispersions. Note that the unpolarized reflectance is the arithmetic mean of the pR and sR.

SEM images were recorded using a high resolution Zeiss Scanning Electron Microscope, with a Gemini 500 column, and using the In Lens, Secondary Electron, and BSD detectors at 5 and 15 kV acceleration voltages respectively, in high vacuum mode. The BSD detector enables materials contrast, crystal orientation and topographic images. Prior to image acquisition an in-situ plasma cleaning process was performed.

The current-voltage characteristic has been recorded using a Keithley 6517 electrometer with incorporated voltage source, and the impedance as function of frequency was measured using a HP 4194A Impedance analyzer.

The hysteresis loops on ferroelectric based capacitor are recorded using a TF2000 ferritester from AixACCT and the capacitance-voltage characteristics are measured using a LCR bridge model Hioki 3536 and the Keithley 6517 electrometer. All the measurements were performed at room temperature.

The pyroelectric signal was recorded with a SR 830 DSP lock-in amplifier, using an J-FET type impedance converter (sample placed on the gate contact, a dc voltage of 7 V applied on the drain contact, and signal collected from a 100 kΩ resistance placed between source contact and ground). The IR source was a laser diode of 30 mW at 800 nm. The beam was modulated electronically using a signal generator from Tektronix, model AFG 3052C.

## Results and discussions

**Fig 2:**
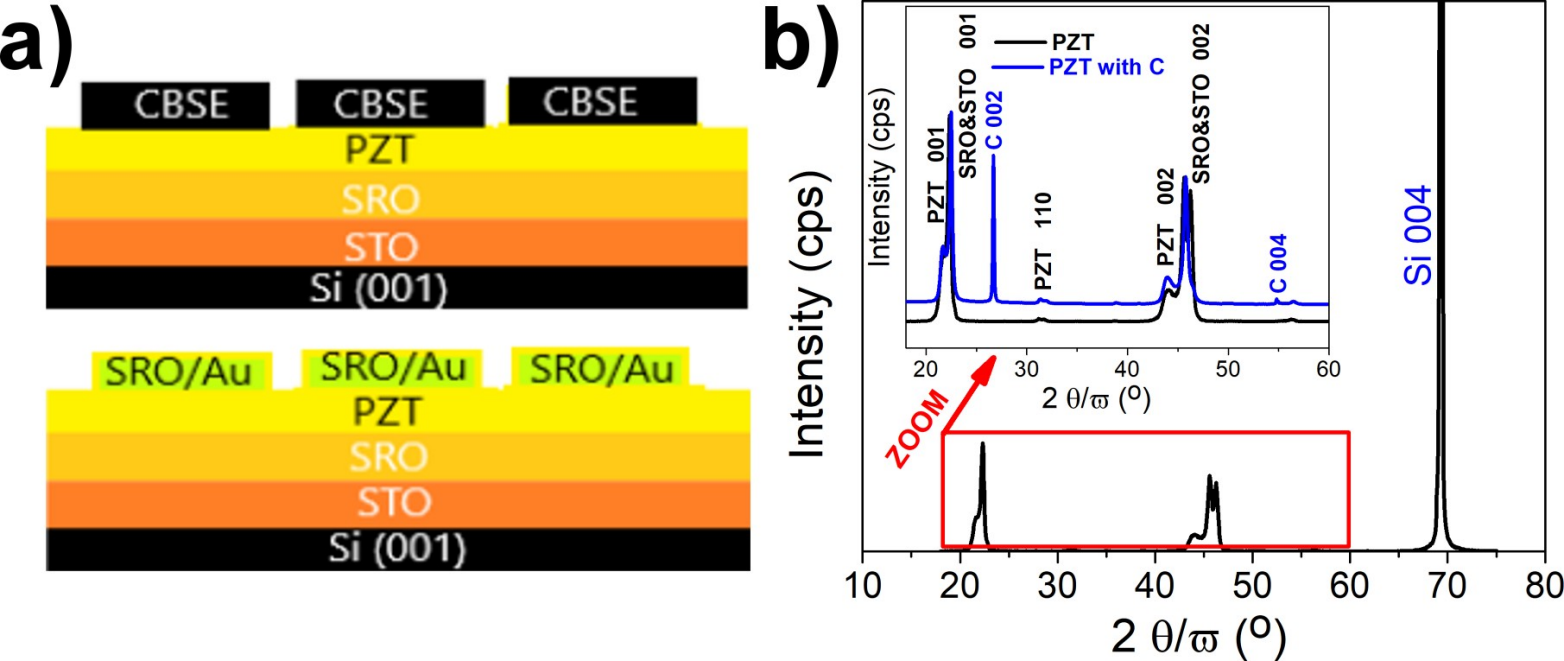
Schematics of the studied structures, showing the component layers (a); XRD 2θ-ω scan for the PZT/SRO/STO structure, with dominant Si peak included (b). The inset shows the XRD patterns for a narrower 2θ range, allowing the evidence of the XRD peaks associate to the deposited PZT, SRO, STO and carbon layers: black line represents the pattern for the PZT/SRO/STO multilayer without CBSE; blue line represents the pattern after deposition of the CBSE layer.

XRD patterns (see Fig 2) did not highlight secondary phases on any of the investigated structures. The strontium titanate (STO) (pseudocubic) film grew epitaxially on the cubic Si substrate (001), favoured by the relationship between the lattice parameters: a_STO_ ~ a_Si_ /√2 (a_STO_ = 3.905 Å, a_Si_ = 5.4307 Å—specific to relaxed lattices). The epitaxial relationship is maintained for the strontium ruthenate (SRO) layer, while the main orientation of the PZT layers is (001) with a very small contribution from the (110) orientation. X-ray diffraction performed after CBSE deposition confirms the existence of highly crystalline form of graphite. The peaks at 26.57° and 54.66^o^ 2θ corresponds to the (002) and (004) reflections in hexagonal graphite with a measured inter-planar spacing of 0.335 nm and 1.67 nm respectively [20].

**Fig 3:**
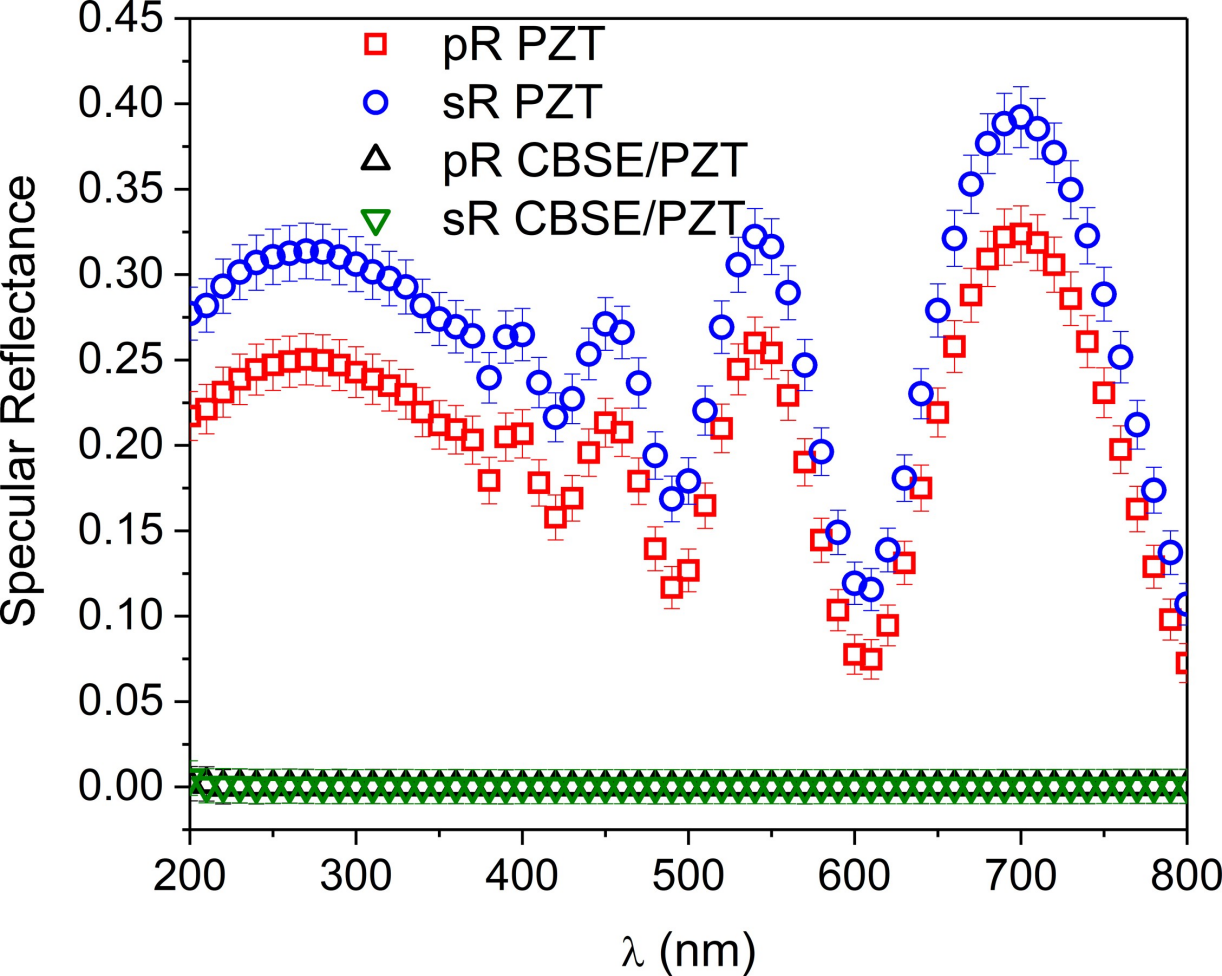
The specular reflectance of PZT layer with and without CBSE (the data and the errors associated to the measurement are given by symbols and bars, respectively). pR and sR stands for p-polarized and s-polarized light, respectively.

The specular reflectance of PZT layer with and without CBSE was measured at 25° incidence angle by spectroscopic ellipsometry, (see Fig 3) (the data and the errors associated to the measurement are given by symbols and bars, respectively). The 25° angle of incidence was chosen to be as close as possible to the laser incidence angle 0^o^ used in case of pyroelectric measurements. As can be observed, the specular reflection of CBSE covered surface is close to zero and this can be associated with the high radiation absorbance in the measured wavelengths range. Due to the fact that our substrate is not transparent the diffuse reflectance and the transmission cannot be measured, for a quantitative absorbance estimation, but taking into account the thickness of CBSE (~3.5 μm) we expect that diffuse reflectance and the transmission components are close to zero [21–24]. Well defined reflection is present in the case of the PZT film uncovered with CBSE. From the interference fringes a thickness of about 390 nm was estimated for the PZT thin film, close to the thickness obtained from cross sectional scanning electron microscopy (SEM) images.

**Fig 4:**
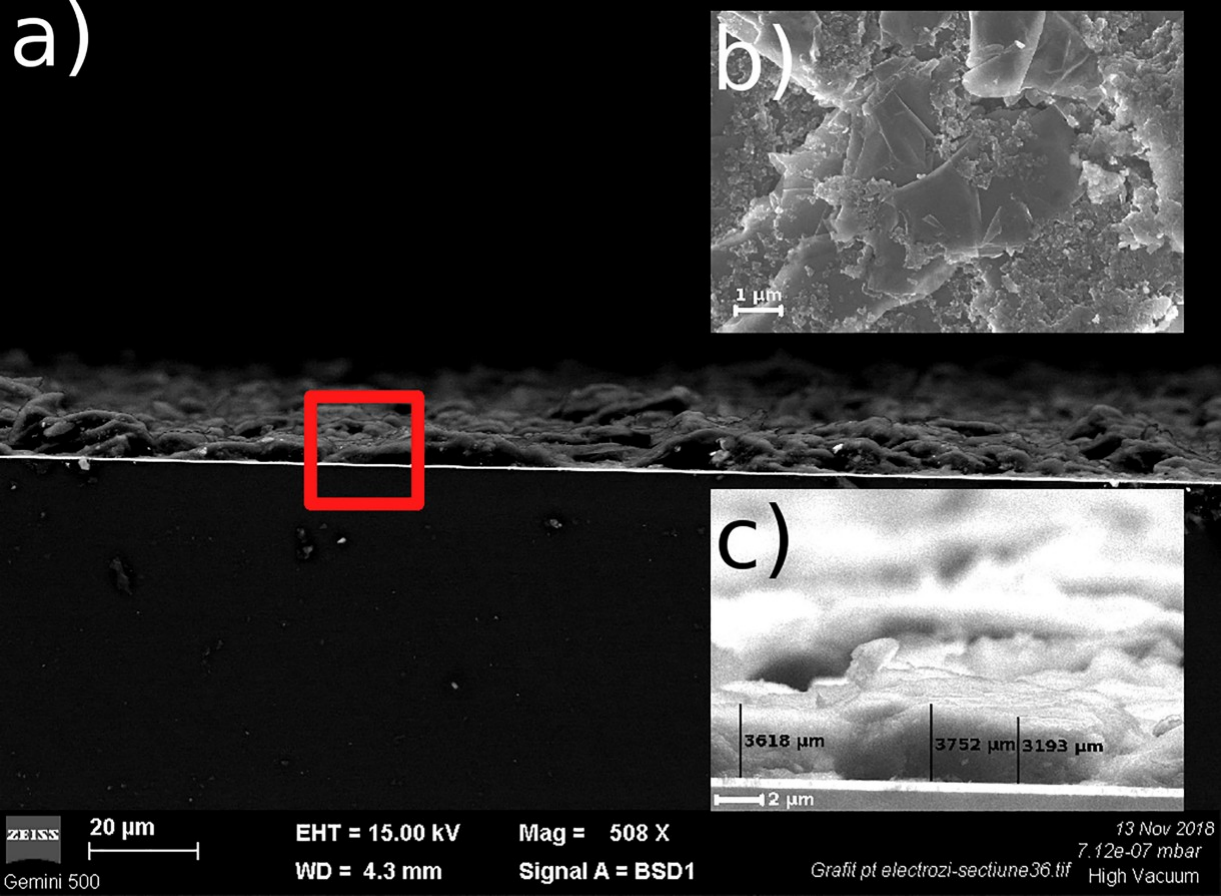
Cross-section SEM image of PZT structure with CBSE electrodes (a) and (c); the surface morphology the CBSE (b).

The surface morphology and the thickness of the CBSE were investigated by SEM, (see Fig 4). From SEM in cross-section (Fig 4(A)) and (Fig 4(C)) we are able to estimate the thickness of the pyroelectric PZT layer to about 370 nm and of the CBSE to approximatively 3.5 μm. In the SEM image, (Fig 4(B)), the CBSE show two different morphologies, one compact layer-stacking morphology, specific for graphite and the second as carbon black agglomerated nanoparticles. This type of morphologies are frequently reported in case of carbon-based structures [25–27].

**Fig 5:**
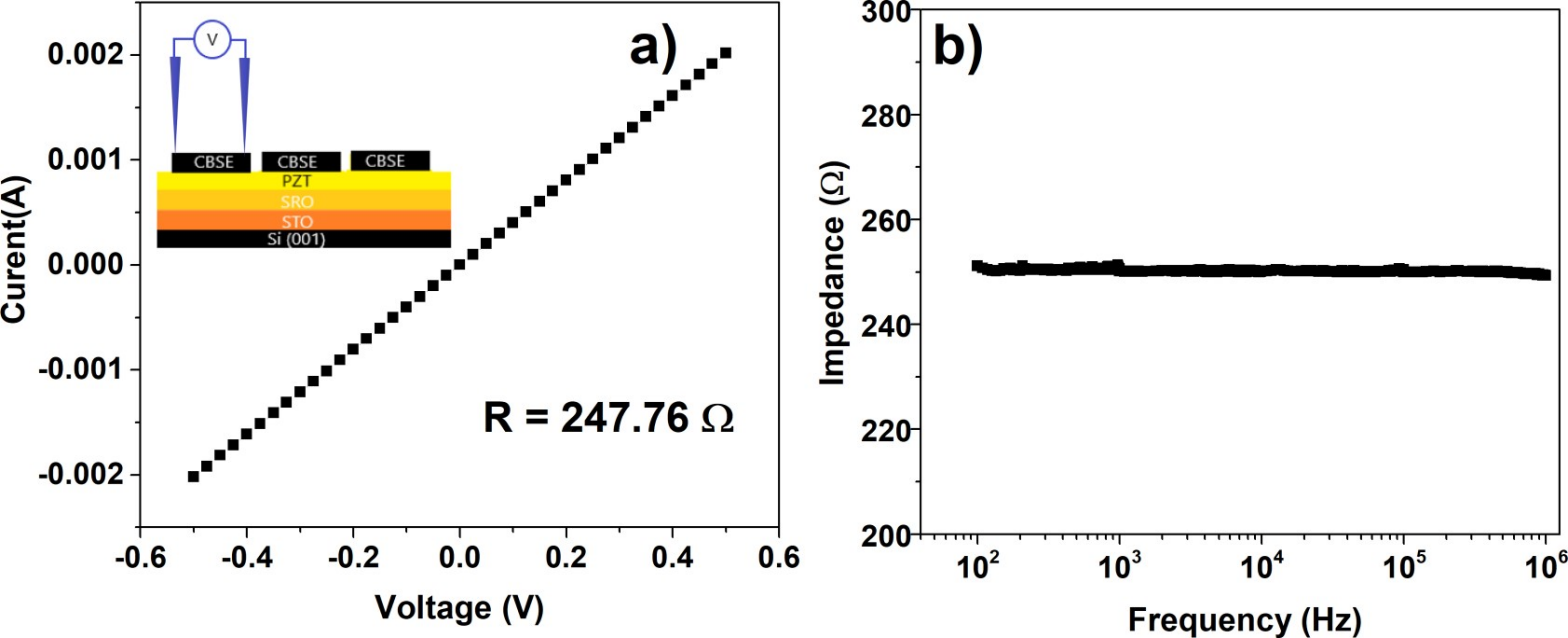
Linear dependence of the current as a function of voltage for CBSE (a); impedance as function of frequency for CBSE (b). The inset shows the in-plane geometry used for measurements.

The electrical properties of the CBSE were analyzed via a typical current-voltage (I-V) characteristic, recorded at room temperature. One has to mention that the measurement was performed on the surface of the CBSE layer using two conductive needles connected to the electrometer. The linear dependence of the current as a function of voltage supports a resistive-like behavior, and from the slope of this linear dependence a value of 247 ohms was estimated for the sheet resistance of CBSE (Fig 5(A)). Also, the impedance presents approximatively constant value of 250 ohms for the frequency range between 100 Hz to 1 MHz (Fig 5(B)). According to the electrical response, we can consider that CBSE may be successfully used as electrode to build capacitor-like structures for pyroelectric applications.

**Fig 6:**
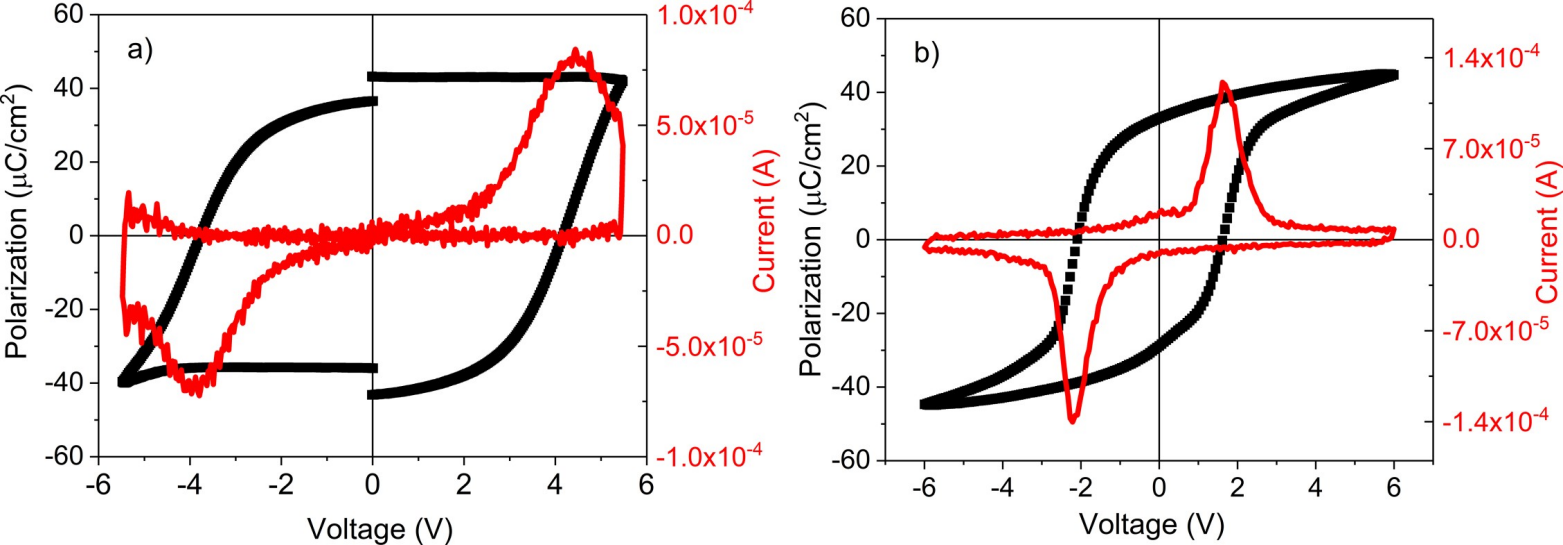
The remnant hysteresis loop for structure with CBSE as top electrode (a); hysteresis loop for structure with SRO/Au as top electrode (b).

After establishing that the CBSE layer acts as an electrode on top of the PZT film, specific measurements were performed on capacitor-like structures in order to compare the ferroelectric and pyroelectric properties of the structures with standard SRO/Au electrode and CBSE, respectively (one capacitor was SRO/PZT/SRO/Au, and the other was SRO/PZT/CBSE). For example, (Fig 6(A)) presents the remnant hysteresis loop recorded for a capacitor structure based on epitaxial PZT 20/80 deposited on Si substrate with STO buffer layer and SRO bottom electrode, with CBSE as top electrode with a delimited area of 0.01 mm^2^. The switching current peaks are well evidenced for both polarities and the remnant polarization value is approximately 40 μC/cm^2^. These ferroelectric characteristics obtained for CBSE are similar to the case of gold covered SRO top electrode (Fig 6(B)). In the case of the CBSE top electrode the current switching peaks are wider and the coercive voltage is increased to 4V compared to 2V in the case of SRO/Au top electrode. The change in the coercive voltage may have several causes, such: as a higher resistance of the CBSE layer when the measurement is performed across the thickness, considering that this layer is much thicker than SRO/Au electrodes (3.5 μm compared to about 100 nm); the significant porosity of CBSE, that may lead to voltage drops across the pores; different properties of the carbon/PZT interface compared to the SRO/PZT interface. All these may lead to an important voltage drop across CBSE compared to SRO/Au, leading to a larger coercive voltage.

**Fig 7:**
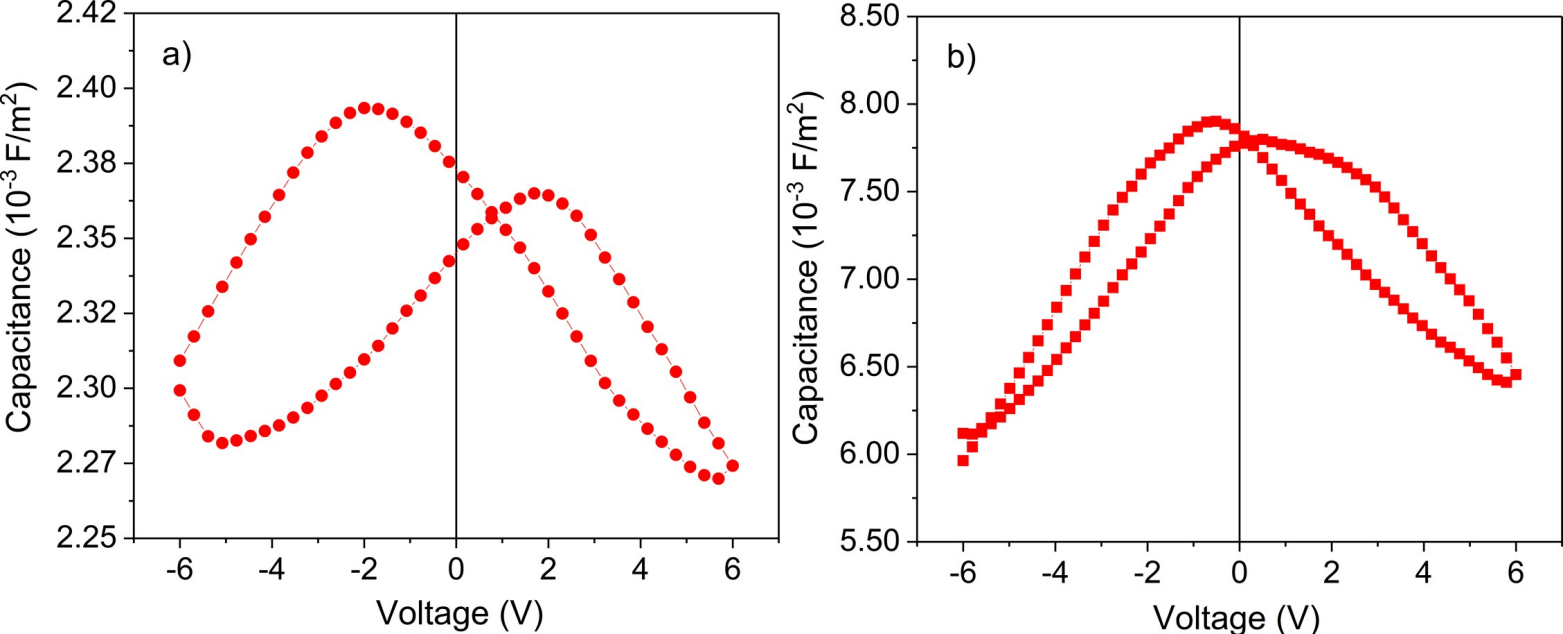
The CV loop recorded for structure: with CBSE as top electrode (a); with SRO/Au as top electrode (b), measured at 100 kHz.

Fig 7(A) presents the capacitance-voltage (C-V) characteristics recorded for a capacitor with CBSE on top. The butterfly shape of the C-V confirms the ferroelectric character of the structure and is similar in shape to the C-V recorded in the case of the capacitor with SRO/Au top electrode, (see Fig 7(B)). However, one can observe that the specific area capacitance is about 3 times larger for top SRO/Au contacts. This can be explained by totally different properties of the carbon/PZT interface compared to the SRO/PZT interface. One knows that metals or oxides with metal behavior like SRO forms Schottky contacts on PZT, the capacitance of the structure being dependent on the metal or oxide used as top electrode [28–30]. It may be that the capacitance associated to the carbon/PZT Schottky contact is significantly smaller than the one associated to the SRO/PZT contact, leading to a smaller overall capacitance of the structure in the assumption of a serial connection between the bulk capacitance of the PZT film (remains the same no matter the material used as top contact) and the capacitance associated to the top Schottky contact (changes when the material used as top electrode is changed).

The results of hysteresis and C-V measurements confirm that CBSE is as good an electrode as classical metallic electrodes for ferroelectric structures.

**Fig 8:**
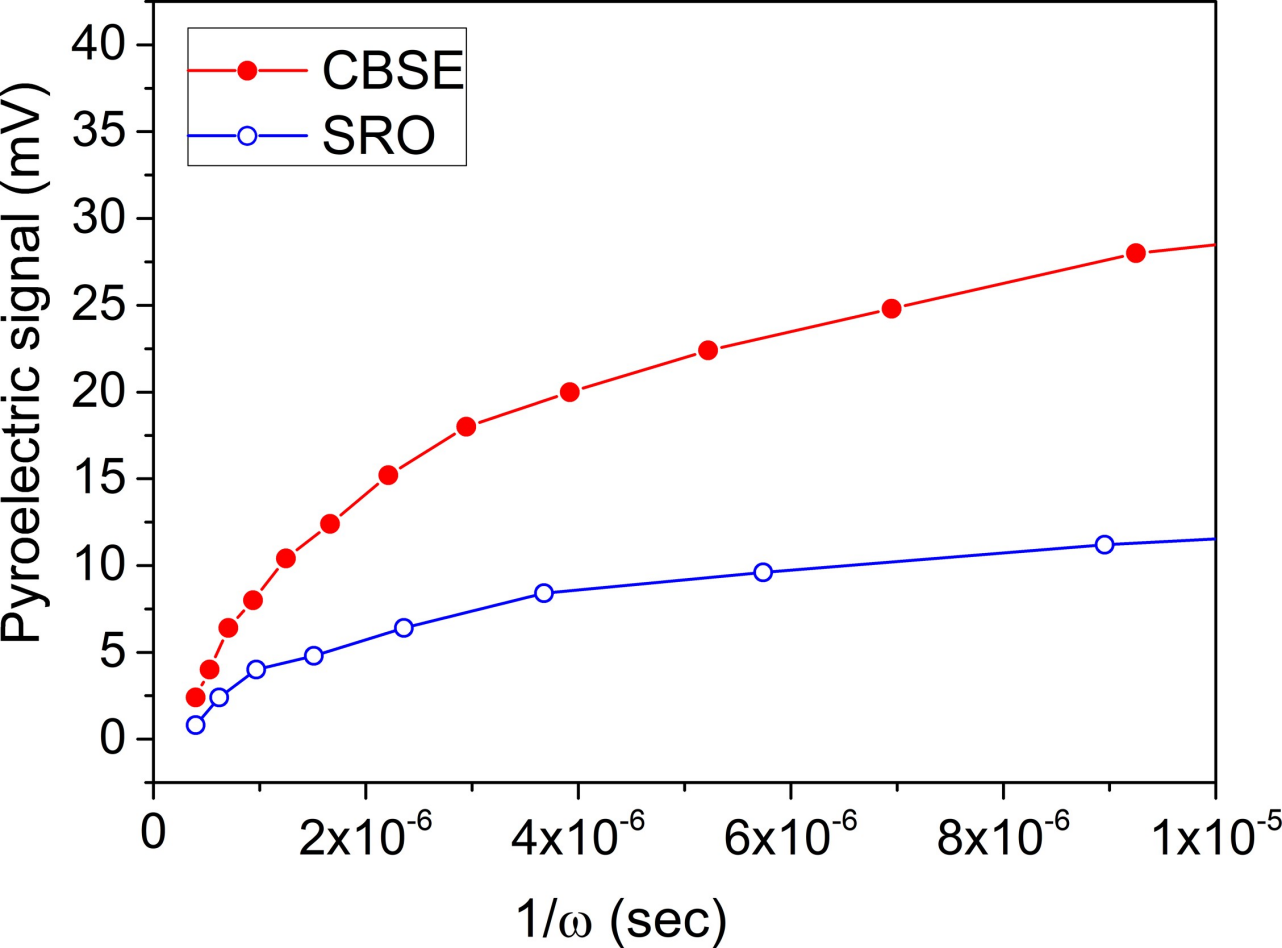
The pyroelectric signal as a function of the reverse of the modulation frequency recorded for structures with top CBSE and SRO/Au electrodes.

The pyroelectric properties of the structures with CBSE and SRO/Au electrodes were investigated in order to assess if CBSE is suitable for pyroelectric applications. In Fig 8 are presented the dependencies of the pyroelectric signal on the reverse of the modulation frequency of the IR beam in the case of the two structures, the one with standard top SRO/Au contact and the one with top CBSE.

The pyroelectric signal recorded on the sample with CBSE is 2.5 times larger than for the sample with SRO/Au electrode at low frequency range. Increasing the modulation frequency of the IR beam, the response of the structure with CBSE decreases faster and converges towards the values obtained in the case of the structure with top SRO/Au electrode. These finding will be explained in the next paragraphs.

The pyroelectric signal in the voltage mode of operation has the following dependence on the frequency of the incident radiation [31]:

$$S=\frac{\omega \eta pAP_{inc}}{2g_{H}G_{e}{\left(1+\omega^{2}\tau_{T}^{2}\right)}^{1/2}{\left(1+\omega^{2}\tau_{e}^{2}\right)}^{1/2}}$$ (1)

where S is the pyroelectric signal, ω is the pulsation of the IR radiation incident on the pyroelectric element, ω = 2πf, with *f* the chopping frequency of the IR radiation, P_*inc*_ is the incident power on the active element, *g* is the emissivity of the electrode exposed to IR radiation, *p* is the pyroelectric coefficient, *A* is the area of the top electrode, *g*_H_ is the heat loss at the top surface (*g*_H_ is estimated as 4σT_0_^3^, where σ is the Stefan- Boltzmann constant and *T*_0_ is the ambient temperature (298 K)), G_*e*_ is the electric conductance, *τ*_*T*_ is the thermal time constant and *τ*_*e*_ is the electrical time constant. The thermal time constant was estimated from the theoretical model described in Ref. [31,32] and the value was aproximatively 55 s.

The voltage responsivity, R_v_, has a similar dependence on the frequency, being defined by the following equation: [33].

$$R_{V}=\frac{S}{P_{inc}}$$ (2)

S is given by Eq (1).

Therefor, at high frequencies, when both (ωτ_T_)^2^ and (ωτ_e_)^2^ are much larger than unity,

S ≈ 1/ ω and the Eq (2) will reduce to:

$$R_{V}=\frac{p}{\varepsilon_{0}\varepsilon_{r}\rho c}\cdot \frac{\eta }{\omega A}$$ (3)

The area of the top electrodes is A = 0.2 mm^2^ and the emissivity η, is ~ 0.98 for CBSE considering that the material is a good absorber and its morphology is compact, and 0.37 for SRO/Au [31]. In this relation, the first fraction represents the figure of merit of the material and it can be estimated from the slope of the representation R_v_ = f (1/ω) [31,33].

Therefore, the figure of merit is defined as M = p/ (ε_0_ ε_r_ ρ c), where: p is the pyroelectric coefficient, c specific heat of the material (350 J/kg·K), ρ the density of the material (7600 kg/m^3^) (ρ∙c, the volume specific heat is 2.6·10^6^ J/m^3^K), ε_r_ the dielectric constant of the material, ε_0_ is the vacuum permittivity (8.85·10^−12^ F/m). For the case of the structures with top CBSE a value of 45.75·10^−3^ m^2^/C was estimated for the figure of merit is, while for the structure with top SRO/Au electrode the figure of merit is estimated to 47.32·10^−3^ m^2^/C. It was expected that both figures of merit to be similar as it is a propriety of the pyroelectric material, respectively of the PZT layer. From figure of merit, we estimate the pyroelectric coefficient for both cases using the following values for the static dielectric constant ε_r_: 300 for the structure with top SRO/Au contact and 100 for the structure with top CBSE. This values were estimated from the specific capacitance value at zero volt as recorded in the C-V characteristics presented in (Fig 7). The difference reflects the difference in the values of the specific capacitance for the two structures and can be explained, as mentioned above, by the fact that the properties of the carbon/PZT and SRO/PZT interfaces are different, including the value of the associated capacitance. Therefore, the pyroelectric coefficients were estimated to 9.73·10^−4^ C/m^2^K for CBSE electrodes and 3.36·10^−4^ C/m^2^K for SRO/Au respectively, reflecting again the possible differences between the top interfaces in the case of the structures with top CBSE or top SRO/Au electrode.

A qualitative explanation can be given assuming the presence of Schottky contacts at the PZT/electrode interfaces [34]. The capacitance measurements suggest a smaller value for the capacitance of the carbon/PZT Schottky contact than for the SRO/PZT Schottky contact. Considering that the capacitance of the bottom SRO/PZT Schottky contact and that of the PZT film remain the same, then the total capacitance of the structure will be lower for the structure with top CBSE, following the equation:

$$C_{total}=\frac{C_{b}C_{PZT}}{C_{b}+C_{PZT}+\frac{C_{b}C_{PZT}}{C_{t}}}$$ (4)

C_b_ is the capacitance of the bottom SRO/PZT interface; C_PZT_ is the capacitance of the PZT layer, and C_t_ is the capacitance of the top PZT electrode interface. As C_b_ and C_PZT_ are the same, when C_t_ is small, as it is the case for top CBSE, the denominator in Eq (4) is increasing and leads to a smaller value for C_total_, while when C_t_ is large, as for top SRO/Au electrode, the denominator is decreasing and leads to a larger value for C_total_. One can assume that the width of the depleted region is smaller for top CBSE, leading to a large C_t_. This fact reflects in a smaller value for the dielectric constant but in a larger value for the pyroelectric coefficient. One has to underline here that this values are extracted for structures with electrodes. On the other hand, similar values for the figure of merit M reflects the pyroelectric quality of the PZT layer independent of the material used for electrodes. One can also assume that a narrower depletion region may favor a more efficient transfer of the charges generated through pyroelectric effect towards electrodes, leading to a larger signal. This couples also with better absorption of the incident IR beam when CBSE is used, due to close to 1 emissivity [35]. All these can explain the 2.5 times increase in the magnitude of the pyroelectric signal when CBSE is used as top contact. In any case, further studies are needed in order to elucidate the impact of different electrode materials on the pyroelectric properties of PZT.

The faster decrease of the pyroelectric signal when CBSE is used as top contact can be explained by the fact that this is much thicker that Au/SRO top contact, leading to a larger thermal time constant τ_T_. Therefore, for modulation frequencies for which the product (ωτ_T_)^2^ is comparable with unity then the signal given by Eq (1) will decrease faster if τ_T_ is larger, and this seems to be the case for top CBSE contact.

## Conclusions

A simple and low cost method was presented to obtain carbon-based electrodes, namely spray deposition using commercial carbon paste dissolved in a commercial solvent and using nitrogen as carrier gas. It was shown that this type of electrodes has good electrical conductivity and high thermal radiation absorption. Using top CBSE for capacitor-like structures based on ferroelectric PZT material we obtained similar ferroelectric properties as for metallic SRO/Au top electrodes. The differences in the values of the dielectric constant and pyroelectric coefficient are attributed to different properties of the top carbon/PZT and SRO/PZT interfaces, most probably related to different width of the depletion region associated to Schottky contacts. The enhancement in the magnitude of the pyroelectric response for top CBSE structure is attribute to a more efficient collection of the charge generated by pyroelectric effects and to a more efficient absorption of the IR light due to larger emissivity. It can be concluded that the spray-coating technique for carbon based electrodes provides a low cost, easy to implement and fast approach to industrial fabrication of various devices such as pyroelectric detectors and energy harvesters, as well as solar cells.
